# The Comparative Effects of White Potato and White Rice Consumption on Measures of Cardiometabolic Health in Individuals with Type 2 Diabetes Mellitus and Features of Metabolic Syndrome

**DOI:** 10.1016/j.cdnut.2025.107518

**Published:** 2025-08-06

**Authors:** Neda S Akhavan, Holly E Clarke, Taylor A Behl, Saiful Singar, Amy P Mullins, Raedeh Basiri, Joshua Kidwell, Bahram H Arjmandi, Claire E Berryman, Robert C Hickner

**Affiliations:** 1 Department of Kinesiology and Nutrition Sciences, University of Nevada Las Vegas, Las Vegas, NV, United States; 2 Department of Health, Nutrition, and Food Sciences, Florida State University, Tallahassee, FL, United States; 3 Center for Advancing Exercise and Nutrition Research on Aging, Florida State University, Tallahassee, FL, United States; 4 Department of Business Administration, Flagler College, St. Augustine, FL, United States; 5 Department of Nutrition and Food Science, George Mason University, Fairfax, VA, United States; 6 Pennington Biomedical Research Center, Louisiana State University, Baton Rouge, LA, United States; 7 Institute of Sports Science and Medicine, Florida State University, Tallahassee, FL, United States

**Keywords:** diabetes, potato, vascular health, cardiovascular, glycemic control

## Abstract

**Background:**

The objective of this study was to compare the effects of daily consumption of white potatoes compared with white rice on cardiometabolic health in individuals with type-2 diabetes (T2D).

**Objective:**

To determine the effects of white potato consumption compared to white rice (a commonly consumed refined grain) on indices of glycemic control and cardiovascular health in individuals with overweight or obesity and T2D.

**Methods:**

In this randomized crossover study, comparative control trial, 24 adults with T2D [45–80 y, body mass index (kg/m^2^) 25–40] consumed baked white potatoes (100 g) or calorie-matched white rice (75 g) daily for 12 wk, separated by a 2-wk washout, with assessments of glycemic control, lipids, inflammation, blood pressure, endothelial function, and body composition at baseline (only 1 baseline visit included as a covariate in statistical analyses), 6 wk, and 12 wk. A linear mixed model was used to assess treatment (potato or rice), time (6 wk or 12 wk), and the treatment-by-time interaction for all outcome variables.

**Results:**

There were no significant (*P* ≤ 0.05) treatment-by-time interactions for any outcome. There was a main effect of treatment (i.e., independent of time) with the potato regimen resulting in lower waist circumference (*P* < 0.0001; 4.5 ± 1.0 cm), percent fat mass (*P* = 0.01; 1.7 ± 0.7%), waist-to-hip ratio (*P* = 0.002; 0.025 ± 0.013), heart rate (*P* = 0.01; 3.1 ± 1.2 bpm), as well as higher percent fat-free mass (*P* = 0.05; 1.4 ± 0.7%) and maximum brachial artery dilation (*P* = 0.05; 0.074 ± 0.037 mm) when compared to the rice regimen. There were significant timepoint effects (i.e., independent of treatment) for increased homeostatic model assessment of β-cell function (*P* = 0.02; 34.3 ± 14.5) and decreased high sensitivity C-reactive protein (*P* = 0.02; 0.08 ± 0.05 *μ*g/mL) and flow-mediated dilation/shear (*P* = 0.03; 4.3 × 10^–5^ ± 3.79 × 10^–5^) during the study.

**Conclusions:**

White potatoes did not negatively affect glycemic indices, vascular health, lipids, or blood pressure compared to white rice and modestly improved body composition and vascular measures. In both groups, over time, there were reductions in flow-mediated dilation/shear stress, β cell function, and high-sensitivity C-reactive protein. Our preliminary results support white potatoes as a substitute for white rice in T2D.

## Introduction

Approximately 38.4 million individuals, or 11.6% of the United States population, have diabetes. Of those with diabetes, 90–95% have type 2 diabetes (T2D), characterized by chronic hyperglycemia and insulin resistance, which increases risk of developing cardiovascular disease (CVD) [1]. Individuals with T2D are known to have accelerated vascular dysfunction, characterized by chronic vasoconstriction that contributes to arterial stiffness in the large arteries and impaired endothelium-dependent vasodilation [[2], [3], [4]]. Moreover, risk factors common to T2D, such as obesity, hypertension, and dyslipidemia, further elevate risk for CVD, as these factors are implicated in the pathogenesis of both conditions [5,6]. Therefore, it is crucial to establish safe, economical, and effective strategies and treatments to improve cardiovascular health in individuals with T2D, as they are a high-risk population.

Diet plays an important role in the prevention and treatment of T2D [7], with particular emphasis placed on the quantity and quality of dietary carbohydrate. Low-carbohydrate, low glycemic index (GI), Mediterranean, and higher-protein diets have been shown to improve glycemic control [8]. In a 12-mo dietary intervention, patients with T2D who were randomly assigned to consume a low GI, high GI, or low-carbohydrate diet had no differences in glycated hemoglobin A1c (HbA1c) concentrations following the 12-mo dietary intervention [9], suggesting high GI foods, in moderate amounts, may be safely incorporated in the diets of T2D patients to provide variety and increase palatability. The United States Dietary Guidelines recommends that total daily carbohydrate intake should be between 45–60% of energy intake for all individuals, with emphasis placed on high fiber-containing carbohydrate sources and limiting refined grains [10,11].

Among these carbohydrate sources, white potatoes (Solanum tuberosum) are nutrient-dense vegetables, containing vitamins (e.g., vitamin C), minerals (e.g., potassium), essential amino acids, fiber, and phytochemicals, but are often avoided by patients with T2D due to their high GI. Potatoes are the richest source of dietary potassium in Western diets, and high potassium diets protect against hypertension [12,13]. Recent epidemiological data have shown that low dietary intake of potassium increases risk for insulin resistance and diabetes [13]. Chlorogenic acid, the major polyphenol in potatoes found in the flesh and mainly in the peel, has been shown to improve insulin sensitivity, lipid profiles, antioxidant status, and reduce blood glucose concentrations and fatty acid synthesis in diabetic mice; therefore, consuming potatoes in whole form will likely provide the greatest benefits for cardiometabolic health [14,15]. Potatoes, particularly when baked or boiled, have been shown to be more satiating when compared to staple grains and sides such as pasta, rice, and corn [16,17]. The majority of the fiber in potatoes is in the form of resistant starch, which has been shown to improve insulin sensitivity, increase satiety, and reduce cholesterol and triglycerides [[18], [19], [20], [21]]. Moreover, a majority of the potato skin contains insoluble fiber (e.g., cellulose, lignans), which has been shown to have cardiometabolic and gut health benefits [22,23]. Despite their starch content, white potatoes offer significant nutritional benefits, including potassium, fiber, and chlorogenic acid, which may support cardiovascular health, highlighting the need to explore the effects of whole potato consumption in individuals with T2D.

To our knowledge, the extent to which daily whole white potato (including the skin) consumption compared to a commonly consumed staple grain (white rice) affects glycemic control and cardiovascular health in individuals with T2D is unknown. Therefore, the objective of this study was to determine the effects of white potato consumption compared to white rice (a commonly consumed refined grain) on indices of glycemic control and cardiovascular health in individuals with overweight or obesity and T2D. In this randomized, crossover, comparative control trial, we explored whether daily consumption of 100 g white potato, compared to an energy- and carbohydrate-matched refined grain (white rice), for 12 wk would have no adverse health effects and would offer modest improvements in glycemic control, vascular function, blood lipid concentrations, body composition, anthropometrics and inflammation in individuals with overweight or obesity and T2D.

## Methods

### Participants

Individuals, including males and postmenopausal females, between the ages of 45 y and 80 y who were overweight or obese [BMI (in kg/m^2^) 25.0–40.0] and had diagnosed T2D (on oral hypoglycemic medication, noninsulin dependent), and were nonfrequent potato consumers (<2 serving of potatoes a week) were recruited from Tallahassee, FL and surrounding areas through campus and community advertisements through flyers, newspaper articles, and public events. Recruitment began in November of 2019 (the COVID-19 pandemic stopped study activities from March to August of 2020) and continued through March of 2022, when the final participant finished the study. Individuals who were taking insulin, diagnosed with CVD, uncontrolled hypertension (≥160/100 mmHg), or other active chronic diseases such as cancer, asthma, glaucoma, thyroid, kidney, liver, and pancreatic disease, or who were pregnant or pre/perimenopausal, or on hormone replacement therapy, were excluded from the study. In addition, individuals who were participating in a weight loss program, heavy smokers (>20 cigarettes per day), and heavy drinkers (>12 alcoholic drinks per week) were excluded from the study. The Florida State University institutional review board approved the study protocol. All participants were provided with detailed information about the study from research staff and then documented their consent by signing the informed consent form prior to participation. This trial was registered at clinicaltrials.gov: NCT04511325.

After an initial telephone screening, potential participants reported to Florida State University (study site) for an in-person visit. At this first visit (screening), potential participants were provided with a verbal and written explanation of the project, and any questions regarding the study were answered by trained research personnel. Then, the individual was asked to sign the informed consent form, followed by confirmation of T2D diagnosis from medical history, medication use, and fasting blood glucose concentrations (≥126 mg/dL). Participants then had anthropometrics and blood pressure assessed, in addition to a 7-d food frequency questionnaire [24] to confirm eligibility. Participants were asked to maintain their usual diet and physical activity pattern throughout the duration of the study.

A CONSORT flowchart of the study enrollment is presented in Supplemental Figure 1. A total of 75 individuals were prescreened (over the phone), with 35 participants screening on-site. Twenty-seven individuals with T2D met inclusion and exclusion criteria and were enrolled and randomly assigned to receive preprepared baked white potato (100 g cooked with skin; 100 kcals) or a calorie-matched refined grain (75 g cooked long-grain white rice; control) daily for 12-wk, separated by a 2-wk washout. Twenty-four participants completed baseline measurements, and 19 participants completed both arms of the study. The overall attrition rate for this 26-wk intervention study was 21%. Some common reasons for participants not finishing the study include the effects of COVID-19, loss of interest, moving to a different city/relocating, or inability to be contacted.

### Study design and intervention

Due to post-COVID recruitment challenges and a reduction in sample size, the primary outcome was changed to percentage flow-mediated dilation (FMD). Therefore, our minimum sample size was calculated with %FMD as the primary outcome variable. Based on previous nutritional intervention studies using FMD, we performed a power calculation expecting to observe a 4.5 ± 4.5% difference in FMD [25]. Thus, with 80–90% power and α = 0.05, we calculated that 20–23 participants would be required in each treatment regimen. This was a 12-wk, randomized, crossover, comparative control clinical trial. At the baseline visit, participants were randomly assigned via computer-generated randomization methods to start 1 of the 2 treatment groups (Table 1): consuming 100 g baked white potato with skin or 75 g calorie-matched refined grain (cooked long-grain white rice) daily for 12 wk. This was followed by a 2-wk washout period, and then the other treatment arm was completed. Participants came for only 1 baseline visit at the beginning of the study and completed 2-wk, 6-wk, and 12-wk study visits with each corresponding treatment (Supplemental Figure 2). The rationale for having a 2-wk washout period between the 12-wk dietary regimen was to facilitate the return to baseline physiological conditions (prior to starting the corresponding dietary regimen) and minimize potential carryover effects, thereby ensuring the independence of each treatment period. The washout period also served as a compliance break to give the participants a break from eating study foods and following study procedures before crossing over to the other treatment arm. The participants were asked to consume the treatment regimen as part of their meals or as a snack throughout the day to contribute to a balanced meal plan, following carbohydrate counting practices [26]. Participants were allowed to add spices and seasonings, but were advised not to add excessive fats to either treatment regimen. Study foods were prepared in the Florida State University Metabolic Kitchen and Diet Assessment Laboratory, where the potato regimen was baked, cooled, and portioned into freezer/microwave safe, single-use containers before freezing (with the rice regimen following a similar protocol after boiling). Participants were asked to come to the study site to pick up their prepackaged study foods every 3-wk. Participants were advised to store the prepackaged food in their freezer and microwave prepackaged study foods daily. Participants were instructed to fill out daily compliance diaries and given 3-d food records to fill out once every 6 wk to assess typical dietary intake throughout the study. At the baseline visit, participants were asked to come to the study site after an overnight fast, 12 h without caffeine, and 24 h without moderate to heavy physical activity. Blood pressure, vascular function, anthropometrics, and body composition were measured, blood was drawn, and a physical activity questionnaire was administered at baseline, in addition to 6-wk and 12-wk visits for both intervention periods (for a total of 5 study visits). After the 2-wk washout period, participants were asked to come to the study site to receive their study food and be given instructions on their new intervention regimen. To monitor compliance, participants were given customized calendars and were asked to record the days they missed consuming the study regimen and return any unused portion for compliance monitoring purposes. Compliance was defined as missing ≤3 doses per week.

**TABLE 1:** Nutrient Composition of 100 g white potato and 75 g long-grain white rice.

	100 g white russet potato with the skin^1^	75 g long-grain white rice^1^
Energy (kcal)	95	98
Fat (g)	0.13	0.21
Total carbohydrates (g)	21	21
Fiber (g)	2.3	0.3
Protein (g)	2.6	2.0
Potassium (mg)	550	26
Vitamin C (mg)	8.3	0

Abbreviation: USDA, United States Department of Agriculture.

1 USDA National Nutrient Database for Standard Reference.

### Anthropometric and body composition assessments

Height without shoes was measured using a wall-mounted stadiometer to the nearest 0.5 cm at baseline. Weight was assessed using a digital scale (Seca Corporation) to the nearest 0.1 kg. BMI was calculated as weight in kilograms per height in meters squared [27]. Midabdominal waist and hip circumferences were measured using a Gulick fiberglass measuring tape with a tension handle (Creative Health Products, Inc.), using the methods from the NHANES IV survey [28]. Bioimpedance spectroscopy (ImpediMed) was used to assess body composition. Briefly, dual-tab electrodes were positioned as recommended by the manufacturer on the wrists and right foot (ulnar and radial styloid processes for wrist and medial and lateral malleoli for the ankle). A painless electrical current passes through the body, measuring resistance and reactance to this electrical current for the determination of total body water (intra and extracellular), fat mass, and lean mass. Two measurements were taken at each study visit and averaged. Body weight, waist and hip circumferences, and body composition by bioimpedance spectroscopy were measured at baseline in addition to 6-wk and 12-wk study visits for each treatment arm.

### Blood pressure, aortic blood pressure, arterial stiffness, and endothelial function

All cardiovascular measurements were performed between 07:00 and 10:00 in the supine position in a quiet temperature-controlled room (23 ± 1^o^C) after an overnight fast, 12 h with no caffeine, and 24 h after the last bout of moderate to heavy physical activity. Blood pressure was measured in the supine position after 10 min of rest, using an oscillometric automated device (Omron HEM-907XL; Omron Healthcare), with 3 separate measurements taken and averaged (≥1 min between each reading). Endothelial function was assessed using the techniques and assessments for FMD, which have been validated by Thijssen et al. [29]. Brachial artery blood flow was measured in the right arm 3–6 cm above the antecubital crease using high-resolution vascular ultrasound (HD11XE; Philips Ultrasound) with a 3–12 MHz linear array transducer, with the ultrasound probe clamped to maintain the image on the same arterial segment. Briefly, a baseline brachial diameter was determined for each participant, with a cuff placed around the forearm for resting assessments. This cuff was then inflated ≤∼250 mmHg for 5 min, and the measurement of vessel diameter began 30 s prior to deflation and 2 min post deflation. Changes in vessel diameter were denoted as percent change from study visits for each treatment arm.

Assessments of aortic blood pressure and arterial stiffness were conducted using brachial-ankle pulse wave analysis (Sphygmo-Cor; AtCor Medical Pty Ltd) and pulse wave velocity (PWV) (VP-2000; Omron Healthcare Inc.) techniques. Appropriate-sized blood pressure cuffs were wrapped around both arms (brachial artery) and ankles (posterior tibial artery). Electrocardiogram electrodes were placed on the forearms, and a heart sound microphone was placed on the left sternal border. The techniques that were used for assessment of aortic blood pressure and arterial stiffness have been validated and have good repeatability [[30], [31], [32], [33]]. Briefly, participants rested for ≥20 min before data collection. Transit time was automatically determined from the time delay between the feet of the pulse waves related to the R-wave of the electrocardiogram. The distance from the carotid and femoral artery was measured with a nonelastic tape measure as a straight line, whereas the distance from the brachial to tibial arteries was calculated automatically according to the participant’s height. PWV was calculated as the distance divided by transit time. Two measurements were collected and averaged at baseline, 6-wk, and 12-wk visits. Heart rate was determined from the electrocardiogram.

### Blood collection and analysis

Fasting finger stick blood collection was performed for measures of fasting blood glucose using a glucometer device (ACCU-CHECK; Roche Diabetes Care), in addition to fasting venous blood for plasma and serum were collected between 07:00 and 10:00 from each participant in vacutainers with appropriate anticoagulants at baseline and 6-wk and 12-wk study visits for each treatment arm. Serum and plasma were separated by centrifuging at 2058 G (3500 × *g*) for 15 min at 4^o^C within 2 h of collection using an IEC CL31R multispeed centrifuge (Thermo Electron Corporation). Samples were then divided into aliquots and stored at –80^o^C until analyses. Plasma lipid profiles (including total cholesterol, HDL, LDL, non-HDL, triglycerides, and VLDL) were assessed using the Piccolo Xpress diagnostic analyzer (Abaxis). Plasma concentrations of oxidized-LDL (Merocida), HbA1c (MyBioSource), and high-sensitive C-reactive protein (hs-CRP) (RnDSystems) and serum concentrations of leptin (Thermo Fisher Scientific), insulin (Thermo Fisher Scientific), and adiponectin (Thermo Fisher Scientific) were measured in duplicate according to the manufacturer’s instructions.

### Statistical analysis

Normality of data was assessed using univariate analysis to quantitatively evaluate skewness and to visually inspect box and probability plots. The statistical analysis was intention-to-treat and used restricted maximum likelihood to account for missing data. A linear mixed model was used to assess treatment (potato or rice), time (6 wk or 12 wk), period (first or second), the treatment ∗ time interaction, and the treatment ∗ period interaction as fixed effects and subject as a random effect on outcome variables. The baseline measurement, taken once at the beginning of the study, was included as a covariate. No carryover effects (i.e., treatment ∗ period interaction) were observed for any variable. *P* ≤ 0.05 was considered statistically significant. We additionally examined corrected *P* values for multiple comparisons, using the False Discovery Rate (FDR) method to control for type I errors due to multiple hypothesis testing. The FDR adjustment was performed using the Benjamini-Hochberg procedure, which ranks the *P* values and adjusts them based on their rank and the total number of tests performed. Adjusted *P* values (q values) below the significance threshold of 0.05 were considered statistically significant. Primary interpretations are based on unadjusted *P* values to highlight exploratory findings, whereas FDR-adjusted results serve as a secondary check to identify robust associations. Statistical analysis software v9.4 (SAS Institute Inc) and RStudio (version 4.3.1) were used for all statistical analyses. FMD was the primary outcome variable; indices of glycemic control, additional indices of vascular function (measures of arterial stiffness, blood pressure, and vessel dilation), blood lipid concentrations, body composition, anthropometrics, and biomarkers of inflammation were secondary outcome variables.

## Results

### Participant characteristics

Baseline characteristic data for participants included in the intention-to-treat analysis are presented in Table 2. Participants demonstrated a mean compliance rate of 90.2% for the potato treatment and 89.1% for the rice comparative control arms.

**TABLE 2:** Baseline characteristics of study participants.

Variable	
Male:female (*n*)	13:11
Age (y)	65 ± 2
Height (cm)	166 ± 4
Weight (kg)	101 ± 8
BMI (kg/m^2^)	31.5 ± 0.8
SBP (mmHg)	136 ± 4
DBP (mmHg)	79 ± 3
HR (bpm)	74.3 ± 2.3
FBG (mg/dL)	146 ± 10
Hypoglycemic medication (% usage)	100
Antihypertensive medication (% usage)	67
Diuretic medication (% usage)	21
Statin medication (% usage)	63
Pain medication (% usage)	29
Caucasian:Hispanic:African American (*n*)	16:1:7

Abbreviations: DBP, diastolic blood pressure; FBG, fasted blood glucose; HR, heart rate; SBP, systolic blood pressure.

### Anthropometrics and body composition

There were no treatment-by-timepoint effects for any anthropometric or body composition measures. There was a main effect of treatment independent of time (least squares mean ± SEM) with potato consumption resulting in lower waist circumference (*P* < 0.0001; 4.5 ± 1.0 cm), percent fat mass (*P* = 0.01; 1.7 ± 0.7%), fat mass (*P* = 0.008; 1.8 ± 0.7 kg), waist-to-hip ratio (*P* = 0.002; 0.025 ± 0.013), as well as higher percent fat-free mass (*P* = 0.05; 1.4 ± 0.7%) when compared to the rice regimen (Table 3).

**TABLE 3:** Anthropometrics and body composition at baseline, 6 wk, and 12 wk after potato and rice treatment regimen.

Variable	Potato (*n* = 19)	Rice (*n* = 21)	Treatment	Timepoint	Treatment-by- timepoint
Weight (kg)					
Baseline	91.2 ± 3.2				
6 wk	90.2 ± 3.9	90.7 ± 3.7	0.20	0.39	0.65
12 wk	90.2 ± 4.5	90.3 ± 3.8			
BMI (kg/m^2^)					
Baseline	31.5 ± 0.8				
6 wk	31.1 ± 0.9	31.1 ± 0.9	0.20	0.36	0.58
12 wk	31.0 ± 1.0	31.0 ± 0.9			
WC (cm)					
Baseline	110 ± 2.5				
6 wk	108 ± 3.2	111 ± 3.0	*<*0.0001^1^	0.31	0.89
12 wk	108 ± 3.6	111 ± 2.9			
HC (cm)					
Baseline	113 ± 1.8				
6 wk	113 ± 1.9	112 ± 1.9	0.78	0.27	0.54
12 wk	113 ± 2.0	114 ± 2.0			
W/H ratio					
Baseline	0.98 ± 0.02				
6 wk	0.97 ± 0.03	0.99 ± 0.03	0.002^1^	0.11	0.66
12 wk	0.95 ± 0.03	0.98 ± 0.02			
FM (kg)					
Baseline	31.2 ± 1.6				
6 wk	28.0 ± 2.0	30.3 ± 2.1	0.008^1^	0.84	0.32
12 wk	29.7 ± 2.1	31.7 ± 2.0			
FM (%)					
Baseline	34.3 ± 1.6				
6 wk	31.8 ± 2.0	33.5 ± 1.8	0.01^1^	0.37	0.45
12 wk	32.9 ± 1.8	34.9 ± 1.7			
FFM (kg)					
Baseline	60.2 ± 2.8				
6 wk	58.6 ± 3.6	59.5 ± 2.8	0.14	0.71	0.18
12 wk	60.9 ± 3.5	59.3 ± 3.0			
FFM (%)					
Baseline	65.8 ± 1.6				
6 wk	67.4 ± 2.1	66.4 ± 1.8	0.05^1^	0.81	0.19
12 wk	67.0 ± 1.8	65.2 ± 1.7			

Data are presented as mean ± SEM. Linear mixed models were used to determine the effects of treatment (potato or rice), time (6-mo or 12-mo), period (1 or 2), the treatment ∗ time interaction, and the treatment ∗ period interaction with baseline included as a covariate on outcome measures.

Abbreviations: FFM, fat-free mass; FM, fat mass; HC, hip circumference; SEM, standard error of the mean; WC, waist circumference; W/H ratio, waist-to-hip ratio.

1 Denotes statistical significance (*P* ≤ 0.05).

### Biomarkers of glycemic control and CVD risk

There were no treatment or treatment-by-timepoint effects for any biomarkers of glycemic control or CVD risk (Table 4). There was a treatment effect trend (*P* = 0.09) for lower fasting blood glucose (9.8 ± 5.7 mg/dL) with the potato compared with the rice regimen. There was a significant (*P* = 0.02) timepoint effect for hs-CRP, with concentrations decreasing (0.08 ± 0.05 μg/mL) from week 6 to week 12 independent of treatment.

**TABLE 4:** Biomarkers of glycemic control, cardiovascular disease risk, and lipid profiles at baseline, 6 wk, and 12 wk after potato and rice treatment regimen.

	Potato (*n* = 19)	Rice (*n* = 21)	Treatment	Timepoint	Treatment-by- timepoint
Glucose (mg/dL)					
Baseline	141 ± 6.2				
6 wk	144 ± 8.6	144 ± 11.8	0.09^1^	0.64	0.19
12 wk	136 ± 9.8	148 ± 13.7			
Insulin (mmol/L)					
Baseline	21.0 ± 0.9				
6 wk	20.4 ± 2.1	21.3 ± 3.3	0.94	0.12	0.89
12 wk	24.2 ± 3.7	24.3 ± 1.7			
HOMA-IR					
Baseline	6.33 ± 0.46				
6 wk	7.11 ± 1.0	7.71 ± 3.32	0.28	0.11	0.74
12 wk	8.23 ± 1.47	8.80 ± 1.05			
HOMA-β					
Baseline	87.0 ± 6.2				
6 wk	85.9 ± 10.1	107 ± 15.9	0.43	0.02^2^	0.99
12 wk	118 ± 17.5	147 ± 20.4			
OX-LDL (u/L)					
Baseline	92.2 ± 6.2				
6 wk	68.8 ± 3.9	70.6 ± 5.7	0.27	0.18	0.62
12 wk	60.7 ± 4.5	67.3 ± 5.6			
Hs-CRP (μg/mL)					
Baseline	0.08 ± 0.02				
6 wk	0.15 ± 0.04	0.17 ± 0.04	0.60	0.02^2^	0.74
12 wk	0.08 ± 0.03	0.08 ± 0.03			
HbA1c (g/mL)					
Baseline	2.61 × 10^–5^ ± 2.4 × 10^–9^		0.45		
12 wk	2.73 × 10^–5^ ± 2.2 × 10^–9^	2.98 × 10^–5^ ± 2.5 × 10^–9^			
Leptin (g/mL)					
Baseline	2.44 × 10^–8^ ± 5.2 × 10^–9^				
6 wk	2.10 × 10^–8^ ± 4.9 × 10^–9^	2.45 × 10^–8^ ± 5.2 × 10^–9^	0.15	0.81	0.15
12 wk	2.07 × 10^–8^ ± 4.7 × 10^–9^	2.79 × 10^–8^ ± 5.7 × 10^–9^			
Adiponectin (g/mL)					
Baseline	4.65 × 10^–6^ ± 5.5 × 10^–7^				
6 wk	5.75 × 10^–6^ ± 1.1 × 10^–6^	5.48 × 10^–6^ ± 8.7 × 10^–7^	0.81	0.13	0.87
12 wk	6.56 × 10^–6^ ± 1.1 × 10^–6^	6.31 × 10^–6^ ± 9.1 × 10^–7^			
Total cholesterol (mg/dL)					
Baseline	154 ± 8.7				
6 wk	167 ± 9.0	168 ± 16.3	0.95	0.37	0.87
12 wk	156 ± 6.9	164 ± 12.2			
HDL (mg/dL)					
Baseline	51.0 ± 3.4				
6 wk	52.4 ± 3.5	50.1 ± 4.3	0.20	0.80	0.92
12 wk	52.4 ± 3.5	53.1 ± 3.6			
Triglycerides (mg/dL)					
Baseline	132 ± 13.1				
6 wk	131 ± 15.1	142 ± 16.3	0.18	0.66	0.24
12 wk	125 ± 14.1	151 ± 17.6			
Non-HDL					
Baseline	103 ±7.9				
6 wk	114 ± 8.3	106 ± 10.1	0.90	0.68	0.37
12 wk	104 ± 6.3	112 ± 11.4			
Total cholesterol/ HDL ratio					
Baseline	3.2 ± 0.24				
6 wk	3.4 ± 0.24	3.3 ± 0.25	0.46	0.56	0.36
12 wk	3.1 ± 0.26	3.3 ± 0.27			
LDL (mg/dL)					
Baseline	76.3 ± 7.8				
6 wk	88.3 ± 8.4	79.3 ± 9.2	0.87	0.59	0.57
12 wk	78.7 ± 5.7	81.4 ± 9.8			
VLDL (mg/dL)					
Baseline	26.3 ± 2.6				
6 wk	26.0 ± 3.0	26.6 ± 3.0	0.22	0.43	0.12
12 wk	24.9 ± 2.8	30.0 ± 3.5			

Data are presented as mean ± SEM. Linear mixed models were used to determine the effects of treatment (potato or rice), time (6-mo or 12-mo), period (1 or 2), the treatment ∗ time interaction, and the treatment ∗ period interaction with baseline included as a covariate on outcome measures.

Abbreviations: HbA1c, hemoglobin A1c; HDL, high-density lipoprotein cholesterol; HOMA-IR, homeostasis model assessment of insulin resistance; HOMA-β, homeostatic model of β cell secretion; hs-CRP, high-sensitivity-C-reactive protein; LDL, low-density lipoprotein cholesterol; OX-LDL, oxidized-low-density lipoprotein cholesterol; SEM, standard error of the mean; VLDL, very low-density lipoprotein cholesterol.

1 Denotes a tendency for statistical significance (*P* < 0.1).

2 Denotes statistical significance (*P* ≤ 0.05).

### Blood pressure, arterial stiffness, and endothelial function

There was a tendency (*P* = 0.08) for PWV (left side only) to have a treatment-by-timepoint interaction, with higher PWV at the 6-wk visit for the rice regimen when compared to potato (0.57 ± 0.31 MpS) as well as an increase at week 12 compared to week 6 for the potato regimen (0.46 ± 0.32 MpS; Table 5). There was a treatment effect for maximum brachial artery dilation (*P* = 0.05; 0.074 ± 0.037 mm; Table 6) in the potato compared with the rice regimen, and a tendency for a treatment effect for resting brachial artery diameter (*P* = 0.07; 0.057 ± 0.030 mm) to be greater in the potato compared with the rice regimen. There was a treatment effect for potato consumption leading to lower heart rate compared to rice consumption (*P* = 0.01; 3.1 ± 1.2 bpm). There was a significant timepoint effect for the FMD/shear stress ratio decreasing from week 6 to week 12, independent of treatment regimen (Table 6).

**TABLE 5:** Hemodynamics and arterial stiffness at baseline, 6 wk, and 12 wk after potato and rice treatment regimen.

Variable	Potato (*n* = 19)	Rice (*n* = 21)	Treatment	Timepoint	Treatment-by-timepoint
SBP (mmHg)					
Baseline	139 ± 4.9				
6 wk	133 ± 3.8	134 ± 4.2	0.25	0.39	0.77
12 wk	134 ± 4.2	137 ± 4.7			
DBP (mmHg)					
Baseline	80.1 ± 2.6				
6 wk	77.9 ± 2.5	78.5 ± 2.3	0.10	0.99	0.33
12 wk	76.5 ± 2.4	79.6 ± 2.6			
HR (bpm)					
Baseline	71.2 ± 2.2				
6 wk	70.6 ± 2.3	73.5 ±2.9	0.01^1^	0.82	0.47
12 wk	69.8 ± 2.9	73.7 ± 2.2			
MAP (mmHg)					
Baseline	99.7 ± 3.0				
6 wk	96.3 ± 2.6	97.1 ± 2.5	0.12	0.64	0.46
12 wk	95.6 ± 2.7	98.6 ± 2.9			
RPWV (MpS)					
Baseline	17.7 ± 0.72				
6 wk	17.8 ± 0.87	18.0 ± 0.79	0.76	0.71	0.67
12 wk	17.9 ± 0.85	18.0 ± 0.80			
LPWV (MpS)					
Baseline	17.5 ± 0.79				
6 wk	17.4 ± 0.92	17.9 ± 0.85	0.71	0.74	0.08^2^
12 wk	17.9 ± 0.89	17.6 ± 0.81			
RABI					
Baseline	1.10 ± 0.02				
6 wk	1.11 ± 0.02	1.13 ± 0.02	0.95	0.19	0.45
12 wk	1.14 ± 0.02	1.14 ± 0.02			
LABI					
Baseline	1.08 ± 0.01				
6 wk	1.10 ± 0.02	1.12 ± 0.02	0.33	0.22	0.51
12 wk	1.12 ± 0.02	1.16 ± 0.02			
Aix (%)					
Baseline	27.4 ± 2.0				
6 wk	24.8 ± 2.5	25.8 ± 2.1	0.80	0.90	0.21
12 wks	26.7 ± 2.1	24.4 ± 2.5			

Data are presented as mean ± SEM. Linear mixed models were used to determine the effects of treatment (potato or rice), time (6-mo or 12-mo), period (1 or 2), the treatment ∗ time interaction, and the treatment ∗ period interaction with baseline included as a covariate on outcome measures.

*Abbreviations: Aix,* augmentation index*; DBP,* diastolic blood pressure; *HR,* heart rate; *LABI,* ankle brachial index-left side; *LPWV,* pulse wave velocity-left side; *MAP,* mean arterial pressure; *RABI,* ankle brachial index-right side; *RPWV,* pulse wave velocity-right side; *SBP,* systolic blood pressure; SEM, standard error of the mean.

1 Denotes statistical significance (*P* ≤ 0.05).

2 Denotes a tendency for statistical significance (*P* < 0.1).

**TABLE 6:** Vascular assessments of endothelial-mediated vasodilation at baseline, 6 wk, and 12 wk after potato and rice treatment regimen.

Variable	Potato (*n* = 19)	Rice (*n* = 21)	Treatment	Timepoint	Treatment-by-timepoint
Resting diameter (mm)					
Baseline	4.28 ± 0.147				
6 wk	4.31 ± 0.150	4.24 ± 0.133	0.07^1^	0.79	0.48
12 wk	4.28 ± 0.141	4.25 ± 0.139			
Resting BF (mL/min)					
Baseline	123 ± 18.7				
6 wk	107 ± 16.0	102 ± 15.1	0.78	0.54	0.99
12 wk	113 ± 17.5	108 ± 10.8			
Max dilation (mm)					
Baseline	4.58 ± 0.150				
6 wk	4.62 ± 0.149	4.58 ± 0.142	0.05^2^	0.23	0.48
12 wk	4.60 ± 0.145	4.51 ± 0.155			
Time to peak (s)					
Baseline	43.6 ± 2.41				
6 wk	45.2 ± 2.25	42.3 ± 2.35	0.12	0.56	0.99
12 wk	46.5 ± 2.95	43.7 ± 2.40			
Change in diameter (mm)					
Baseline	0.303 ± 0.0233				
6 wk	0.306 ± 0.0220	0.306 ± 0.0219	0.27	0.14	0.33
12 wk	0.297 ± 0.0230	0.262 ± 0.0259			
FMD (%)					
Baseline	7.28 ± 0.665				
6 wk	7.30 ± 0.609	7.31 ± 0.600	0.27	0.19	0.31
12 wk	7.17 ± 0.646	6.32 ± 0.694			
Shear rate (AUC)					
Baseline	33,891 ± 2724				
6 wk	33,554 ± 2915	34,065 ± 3510	0.55	0.73	0.69
12 wk	33,449 ± 2366	35,473 ± 2901			
FMD/shear					
Baseline	0.000229 ± 0.0000213				
6 wk	0.000258 ± 0.0000353	0.000245 ± 0.0000271	0.14	0.03^2^	0.53
12 wk	0.000227 ± 0.0000220	0.000190 ± 0.0000202			

Data are presented as mean ± SEM. Linear mixed models were used to determine the effects of treatment (potato or rice), time (6-mo or 12-mo), period (1 or 2), the treatment ∗ time interaction, and the treatment ∗ period interaction with baseline included as a covariate on outcome measures.

Abbreviations: AUC, area under the curve; BF, blood flow; FMD, flow-mediated dilation; SEM, standard error of the mean.

1 Denotes a tendency for statistical significance (*P* < 0.1).

2 Denotes statistical significance (*P* ≤ 0.05).

### Dietary intake, sleep, and physical activity records

There was a tendency (*P* = 0.06) for a treatment-by-timepoint interaction for saturated fat consumption, with greater saturated fat intake in the potato regimen (14.0 ± 5.3 g) at the 6-wk visit when compared to the rice regimen (Supplemental Table 1). There was a tendency (*P* = 0.09) for a timepoint effect for carbohydrate intake increasing from week 6 to week 12 (36.5 ± 14 g) independent of treatment. There was a treatment effect for subjects participating in greater hard activity on weekend days (*P* = 0.05; 20 ± 10 min) during the rice compared with the potato arm (Supplemental Table 2). There were no other treatment-by-time, treatment, or timepoint effects for dietary intake, sleep, or physical activity measures.

### Secondary analysis FDR correction using the Benjamini-Hochberg procedure

There continued to be a main effect of treatment with potato consumption resulting in lower waist circumference (*P* = 0.01; 4.5 ± 1.0 cm) when compared to the rice regimen (Supplemental Table 3). All tendencies and other significant treatment and timepoint effects lost statistical significance after the correction was applied (Supplemental Tables 3–6).

## Discussion

To our knowledge, this is the first clinical trial examining the impact of white russet potato consumption compared to white rice consumption on anthropometrics, body composition, endothelial function, arterial stiffness, and indices of glycemic control and inflammation in adults who are overweight or obese with T2D. The major findings of this study are that the addition of 100 g of white potatoes to the habitual diet did not appear to have negative effects on measured outcomes when compared to 75 g white rice, and may offer some protective effects on waist circumference, fat and fat-free mass, waist-to-hip ratio, heart rate, fasting blood glucose, and maximum brachial artery dilation when compared to the rice regimen. Both interventions over time, the addition of 100 g white potatoes or the addition of an isocaloric amount of white rice in the diet, contributed to reductions in FMD/shear, β cell function, and hs-CRP in adults with T2D.

When adjusting for multiple comparisons using the FDR correction, the statistical significance of several outcomes was lost, with only the reduction in waist circumference remaining statistically significant. This result highlights the robustness of the waist circumference finding, suggesting a potential clinical relevance of this outcome to individuals with T2D. The other observed significant effects, losing statistical significance after FDR correction, emphasize the exploratory nature of these findings. The focus on raw *P* values in this study reflects an intent to identify patterns and generate hypotheses about the cardiometabolic effects of potato consumption compared to white rice. Although FDR correction reduces the likelihood of false-positive results, it may also increase the risk of false negatives, potentially obscuring meaningful trends in the data, particularly in studies with limited sample sizes. Presenting results both with and without FDR correction allows for a refined interpretation, balancing statistical rigor with the exploration of cardiometabolic outcomes assessed in the study. The results from this study provide evidence that white potatoes can be healthfully incorporated in the diet of individuals with T2D, when substituted for other foods with a high glycemic load, such as long-grain white rice.

Although there were no treatment-by-timepoint interactions for any study outcomes, there were treatment effects (i.e., independent of timepoint) for several measures. In a randomized controlled trial, Shih et al. [34] observed a 5% decrease in BMI from white sweet potato consumption (in a lyophilized form used as a meal replacement) compared to a control (nonpotato) diet for 8 wk in individuals who were overweight. Results from our study indicated that participants following the potato regimen had lower waist circumference, waist-to-hip ratio, and fat mass when compared to white rice within our free-living population of adults with T2D. Moreover, no differences in BMI between treatment arms were observed in our study, which is consistent with others examining purple or potato-based side dishes compared to no potatoes, white potatoes, or a refined grain side dish [[35], [36], [37]].

This is the first study to examine the effects of potato consumption on endothelial function, specifically using FMD, within any population. There was a significant timepoint effect for FMD/shear stress decreasing in both groups from 6 wk to 12 wk (12% for the potato and 23% for the rice regimen, respectively), indicating both groups had a similar degree of impairment in endothelial function during the study. However, participants had lower maximal brachial artery dilation during the rice regimen as compared to the potato regimen, indicating the potato regimen may have had some protective effect on vascular health in this population with T2D. Moreover, the value for FMD/shear stress at 12 wk with the potato regimen was at a similar level as the baseline value, whereas the rice regimen at 12-wk had a greater decrease below baseline measures.

In a crossover clinical trial by Johnston et al. [36], healthy individuals were given steamed or baked potatoes (red, white, and gold varieties) with the skin as a side dish with their main meal or a calorie-matched refined grain side dish for 4 wk. Results from Johnston et al. [36] showed no significant differences in blood pressure or PWV. Tsang et al. [37], in a crossover clinical trial, observed a significant (*P* = 0.001) reduction in PWV from 2 wk of purple potato consumption when compared to white potatoes, with no significant changes in indices of glycemic control, lipid profiles, or blood pressure for either treatment. Findings from our study did not show improvements in PWV, similar to those of Tsang et al. [37] for white potato consumption in a healthy population. Our results showed a tendency for PWV (left side only) to have a treatment-by-timepoint interaction, with higher PWV at the 6-wk visit for the rice regimen when compared to potato (0.57 ± 0.31 MpS), as well as an increase from 6-wk to 12-wk for the potato regimen (0.46 ± 0.32 MpS). Although there was a tendency, these results are not of clinical significance as differences of <1.0 MpS, particularly with no changes in blood pressure and heart rate (observed in our study), are not associated with increased CVD risk and can be associated with normal or technical variability in the measurement process [38,39]. In another crossover study by Vinson et al. [35], hypertensive participants were given purple potatoes twice daily compared with no potatoes in a 4-wk crossover clinical trial. After 4 wk of purple potato consumption, diastolic blood pressure significantly decreased by 4.3% (4 mmHg reduction). Results from our study show diastolic blood pressure was 3.9% (3 ± 1.6 mmHg) lower (nonsignificant) at the 12-wk study visits in the potato compared to the rice arm. Our results also showed a lower heart rate for the potato arm when compared to the rice arm. Potatoes are a rich source of potassium, which may have contributed to the modest positive effects on resting heart rate and blood pressure when compared to the rice regimen.

There was a trend for lower fasting blood glucose concentrations observed with the potato regimen when compared to the rice regimen, which may be of clinical importance [40]. Other investigators [[35], [36], [37]] have observed no differences in fasting blood glucose concentrations between potato consumption and controls (including white potatoes, rice, and no potatoes as controls), in relatively healthy populations. Although insulin and HOMA-IR did not have statistically significant differences in either group, there was a significant increase in homeostatic model assessment of β-cell function from 6-wk to 12-wk in both groups, which was a result of increased insulin concentrations observed with both treatment regimens. We cannot explain why this occurred, yet the increase in insulin over time occurred independently of the dietary intervention. This can suggest that the rise in insulin may be due to natural metabolic adaptations in addition to the increased overall carbohydrate intake (noted from 3-d food records), which had a tendency for a timepoint effect, increasing in both groups rather than the specific effect of either potatoes or rice. Results for HbA1c concentrations in our study showed no significant differences after 12 wk of the potato or rice regimen. This finding is consistent with Vinson et al. [35], who similarly saw no changes in HbA1c after 4-wk supplementation of purple majesty compared to no potatoes in subjects with hypertension.

With regards to biomarkers related to CVD risk, there were no significant treatment-by-timepoint interactions observed for oxidized-LDL, leptin, or adiponectin in our study. We cannot explain the elevated concentrations of hs-CRP observed in both the potato and the rice regimens at 6 wk, but values in both groups returned to baseline levels at 12 wk. Results from our study are consistent with those of Johnston et al. [36], who have observed no differences in hs-CRP with potato consumption compared to refined grains [37]. Moreover, lipid concentrations assessed in the current study were not significantly different between the white potato and rice regimens. Clinical studies have shown similar results with no significant differences in lipid profiles from potato consumption (including purple, red, and white potatoes) in healthy or hypertensive populations [[35], [36], [37]].

Our study was strengthened by the fact that it was a randomized crossover clinical trial with a 12-wk intervention. The inclusion of a midpoint (6-wk) study visit allowed us to assess potential differences in outcome variables prior to the 12 wk of each treatment arm. Another strength of our study was the preparation of baked potatoes with the skin, as baking retains the most nutrients and potato peels are a rich source of fiber [41]. In a crossover study from Patterson et al. [42], chilled or boiled potatoes (without the skin) were given to females with elevated fasting glucose and insulin and then assessed for indices of glycemic control and satiety. Results from that study demonstrated that consuming chilled potatoes contributed to significant reductions in postprandial glucose and insulin concentrations. For our current study, we prepared both treatment regimens in a metabolic kitchen, where both potatoes and rice were cooled before freezing, which may have increased retrogradation and lowered the GI of both foods, although when reheated, some of the retrogradation is not maintained [[43], [44], [45]]. Intake of chilled and prefrozen potatoes with the skin warrants further research in individuals with T2D.

Limitations of the current study include our reduced sample size due to COVID-related delays to recruitment efforts, particularly in this high-risk population. A larger sample size may be needed to observe differences in a majority of the parameters assessed; hence, the results of this study are preliminary. Although participants were asked to maintain their usual diet and physical activity patterns throughout the study duration, we did observe a tendency for a timepoint effect for carbohydrate intake increasing in both groups from 6-wk to 12-wk study visits, a tendency for a treatment-by-timepoint interaction for greater saturated fat consumption with the potato regimen at the 6-wk visit when compared to the rice regimen, and a treatment effect for subjects participating in greater hard activity on weekend days in the rice arm compared with potato arm. Another potential limitation is the nature of single food clinical trials within free-living populations, which do not control for unintentional dietary changes. Future clinical trials should look at potato intake with the following specific dietary patterns. Although timepoint effects were observed in both groups, showing reductions in FMD/shear and β cell function, we acknowledge the need for future research to further examine these outcomes and the potential for both regimens to have deleterious effects. Yet, we believe it would be misleading to suggest potential harm in the absence of evidence supporting such a conclusion at this time.

Another possible limitation of the study was the medication variability in adults with T2D, as all participants had to be on ≥1 hypoglycemic medication for inclusion, but there were no restrictions on antihypertensive medications or statins. Therefore, a larger sample size may be needed to observe differences in many of the parameters assessed. It is possible that the effects of the medications may have masked improvements and reduced the sensitivity to dietary intervention in some of the assessments related to blood pressure and lipid profiles. Additionally, baseline measures of blood pressure and lipid concentrations were normal or only slightly elevated, whereas improvements may have been seen in these parameters if the participants had elevated baseline levels. Although a strength of our study was conducting a free-living trial to reflect real-world conditions providing participants with flexibility in how they incorporated the potato or rice regimen into their diets following education on carbohydrate counting and incorporating both regimens as a snack or side dish, we acknowledge that we did not examine if participants replaced either regimen with foods they normally consume or not, which may potentially limit generalizability. Moreover, due to the nature of our free-living trial design, the use of nutritional/herbal supplements, pro/prebiotics, and other bioactive compounds was not controlled, which is another limitation of our study. Future studies could focus more on examining the effects of a dietary treatment, potential dietary replacements, and controlling for supplement intake/usage to better understand the context of these findings.

In conclusion, our study supports the consumption of baked white potatoes with the skin, when consumed in place of foods with a high glycemic load, such as long-grain white rice, in populations with T2D.

## Author contributions

The authors’ responsibilities were as follows – NSA, RCH, CEB, BHA: designed the research; NSA, CEB: analyzed the data; NSA, RCH, CEB: interpreted the data; NSA: wrote the manuscript; NSA, RCH: had primary responsibility for the final content; and all authors: read and approved the final manuscript.

## Data availability

Data described in the manuscript will be made available upon request, pending application and appropriate regulatory approval.

## Funding sources

This study was supported by the Alliance for Potato Research and Education.

## Conflict of interest

NSA reports a relationship with the Alliance for Potato Research and Education that includes speaking and lecture fees. All other authors report no conflicts of interest.
