# Supplementary material for: The Comparative Effects of White Potato and White Rice Consumption on Measures of Cardiometabolic Health in Individuals with Type 2 Diabetes Mellitus and Features of Metabolic Syndrome
Source: Curr Dev Nutr. 2025 Aug 6;9(9):107518. doi: 10.1016/j.cdnut.2025.107518 (PMC12414893; doi:10.1016/j.cdnut.2025.107518)
Supplement: Multimedia component 1 [file mmc1.docx]

**Supplementary Table 1.** Dietary Intake at Baseline, 6 and 12 Weeks After Potato and Rice Treatment Regimen

| **Variable** | **Potato**  **(n=10)** | **Rice**  **(n=10)** | **Treatment** | **Timepoint** | **Treatment-by-**  **Timepoint** |
| --- | --- | --- | --- | --- | --- |
| ***Total (kcal)*** |  |  |  |  |  |
| Baseline | 2213±213 | |  |  |  |
| 6 Weeks | 2120± 170 | 2063± 147 | 0.59 | 0.32 | 0.41 |
| 12 Weeks | 2146± 252 | 2397±231 |  |  |  |
| ***FAT (g)*** |  |  |  |  |  |
| Baseline | 103±13 | |  |  |  |
| 6 Weeks | 101±15 | 76±6.6 | 0.16 | 0.64 | 0.24 |
| 12 Weeks | 92±11 | 96.9±5.8 |  |  |  |
| ***CHO (g)*** |  |  |  |  |  |
| Baseline | 244±23 | |  |  |  |
| 6 Weeks | 214± 18 | 239±21 | 0.16 | 0.09^#^ | 0.69 |
| 12 Weeks | 248± 33 | 278±34 |  |  |  |
| ***PRO (g)*** |  |  |  |  |  |
| Baseline | 81.5±6.1 | |  |  |  |
| 6 Weeks | 89.9±8.7 | 99.5±18.0 | 0.37 | 0.97 | 0.84 |
| 12 Weeks | 87.0±14.2 | 105±15.0 |  |  |  |
| ***SATFAT (g)*** |  |  |  |  |  |
| Baseline | 33.6±6.8 | |  |  |  |
| 6 Weeks | 36.8±6.9 | 23.5±3.0 | 0.11 | 0.86 | 0.06^#^ |
| 12 Weeks | 29.6±4.8 | 34.1±5.8 |  |  |  |
| ***FIBER (g)*** |  |  |  |  |  |
| Baseline | 16.7±2.3 | |  |  |  |
| 6 Weeks | 16.7±3.1 | 15.6±2.4 | 0.69 | 0.10 | 0.67 |
| 12 Weeks | 20.7±4.3 | 21.9±3.5 |  |  |  |

Data are presented as mean ± SEM. Linear mixed models were used to determine the effects of treatment (potato or rice), time (6- or 12-mo), period (1 or 2), the treatment*time interaction, and the treatment*period interaction with baseline included as a covariate on outcome measures. Kilocalories (KCAL); carbohydrates (CHO); protein (PRO); saturated fat (SATFAT). *Denotes statistical significance (*P*≤0.05). ^#^Denotes a tendency for statistical significance (*P*<0.1).

**Supplementary Table 2.** Sleep and Physical Activity Records at Baseline, 6 and 12 Weeks After Potato and Rice Treatment Regimen

| **Variable** | **Potato**  **(n=19)** | **Rice**  **(n=21)** | **Treatment** | **Timepoint** | **Treatment-by-**  **Timepoint** |
| --- | --- | --- | --- | --- | --- |
| ***Sleep (Weekday, h:min)*** |  |  |  |  |  |
| Baseline | 6:47 ± 00:13 | |  |  |  |
| 6 Weeks | 6:51 ± 00:16 | 6:44 ± 00:13 | 0.81 | 0.55 | 0.81 |
| 12 Weeks | 6:37 ± 00:16 | 6:40 ± 00:23 |  |  |  |
| ***Sleep (Weekend. h:min)*** |  |  |  |  |  |
| Baseline | 7:02 ± 00:15 | |  |  |  |
| 6 Weeks | 6:50 ± 00:16 | 7:11 ± 00:16 | 0.14 | 0.63 | 0.90 |
| 12 Weeks | 6:56 ± 00:17 | 7:16 ± 00:15 |  |  |  |
| ***Moderate Activity (Week)*** |  |  |  |  |  |
| Baseline | 2:41 ± 00:36 | |  |  |  |
| 6 Weeks | 2:51 ± 00:24 | 4:02 ± 00:58 | 0.28 | 0.93 | 0.33 |
| 12 Weeks | 3:24 ± 00:43 | 3:33 ± 00:40 |  |  |  |
| ***Moderate Activity (Weekend)*** |  |  |  |  |  |
| Baseline | 1:25 ± 00:19 | |  |  |  |
| 6 Weeks | 1:41 ± 00:32 | 2:11 ± 00:30 | 0.30 | 0.43 | 0.90 |
| 12 Weeks | 1:13 ± 00:25 | 1:53 ± 00:25 |  |  |  |
| ***Hard Activity (Week)*** |  |  |  |  |  |
| Baseline | 1:02 ± 00:25 | |  |  |  |
| 6 Weeks | 00:41± 00:19 | 00:48 ± 00:23 | 0.28 | 0.50 | 0.31 |
| 12 Weeks | 00:35 ± 00:19 | 1:25 ± 00:36 |  |  |  |
| ***Hard Activity (Weekend)*** |  |  |  |  |  |
| Baseline | 00:19 ± 00:07 | |  |  |  |
| 6 Weeks | 00:06 ± 00:04 | 00:36 ± 00:18 | 0.05* | 0.35 | 0.28 |
| 12 Weeks | 00:08 ± 00:06 | 00:16 ± 00:07 |  |  |  |
| ***Very Hard Activity (Week)*** |  |  |  |  |  |
| Baseline | 00:23 ± 00:16 | |  |  |  |
| 6 Weeks | 00:05 ± 00:05 | 00:09 ± 00:09 | 0.69 | 0.20 | 0.67 |
| 12 Weeks | 00:00 ± 00:00 | 00:00 ± 00:00 |  |  |  |
| ***Very Hard Activity (Weekend)*** |  |  |  |  |  |
| Baseline | 00:20 ± 00:14 | |  |  |  |
| 6 Weeks | 00:09 ± 00:09 | 00:09 ± 00:09 | 0.98 | 0.17 | 0.98 |
| 12 Weeks | 00:00 ± 00:00 | 00:00 ± 00:00 |  |  |  |

Data are presented as mean ± SEM. Linear mixed models were used to determine the effects of treatment (potato or rice), time (6- or 12-mo), period (1 or 2), the treatment*time interaction, and the treatment*period interaction with baseline included as a covariate on outcome measures. *Denotes statistical significance (*P*≤0.05). ^#^Denotes a tendency for statistical significance (*P*<0.1).

**Supplementary Table 3.** Anthropometrics and Body Composition at Baseline, 6 and 12 Weeks After Potato and Rice Treatment Regimen.

| Variable | Potato (n=19) | Rice  (n=21) | Treatment | Timepoint | Treatment-by- Timepoint |
| --- | --- | --- | --- | --- | --- |
| *Weight (kg)* |  |  |  |  |  |
| Baseline | 91.2±3.2 | |  |  |  |
| 6 Weeks | 90.2± 3.9 | 90.7± 3.7 | 0.69 | 0.81 | 0.94 |
| 12 Weeks | 90.2± 4.5 | 90.3± 3.8 |  |  |  |
| *BMI (kg/m^2^)* |  |  |  |  |  |
| Baseline | 31.5±0.8 | |  |  |  |
| 6 Weeks | 31.1± 0.9 | 31.1± 0.9 | 0.69 | 0.79 | 0.91 |
| 12 Weeks | 31.0± 1.0 | 31.0± 0.9 |  |  |  |
| *WC (cm)* |  |  |  |  |  |
| Baseline | 110±2.5 | |  |  |  |
| 6 Weeks | 108± 3.2 | 111± 3.0 | 0.01* | 0.77 | 0.96 |
| 12 Weeks | 108± 3.6 | 111± 2.9 |  |  |  |
| *HC (cm)* |  |  |  |  |  |
| Baseline | 113±1.8 | |  |  |  |
| 6 Weeks | 113± 1.9 | 112± 1.9 | 0.94 | 0.73 | 0.90 |
| 12 Weeks | 113± 2.0 | 114± 2.0 |  |  |  |
| *W/H Ratio* |  |  |  |  |  |
| Baseline | 0.98±0.02 | |  |  |  |
| 6 Weeks | 0.97± 0.03 | 0.99±0.03 | 0.12 | 0.69 | 0.94 |
| 12 Weeks | 0.95± 0.03 | 0.98± 0.02 |  |  |  |
| *FM (kg)* |  |  |  |  |  |
| Baseline | 31.2±1.6 | |  |  |  |
| 6 Weeks | 28.0± 2.0 | 30.3± 2.1 | 0.25 | 0.96 | 0.77 |
| 12 Weeks | 29.7± 2.1 | 31.7± 2.0 |  |  |  |
| *FM (%)* |  |  |  |  |  |
| Baseline | 34.3±1.6 | |  |  |  |
| 6 Weeks | 31.8± 2.0 | 33.5±1.8 | 0.25 | 0.79 | 0.85 |
| 12 Weeks | 32.9± 1.8 | 34.9± 1.7 |  |  |  |
| *FFM (kg)* |  |  |  |  |  |
| Baseline | 60.2±2.8 | |  |  |  |
| 6 Weeks | 58.6± 3.6 | 59.5± 2.8 | 0.69 | 0.94 | 0.69 |
| 12 Weeks | 60.9± 3.5 | 59.3± 3.0 |  |  |  |
| *FFM (%)* |  |  |  |  |  |
| Baseline | 65.8±1.6 | |  |  |  |
| 6 Weeks | 67.4± 2.1 | 66.4± 1.8 | 0.62 | 0.94 | 0.69 |
| 12 Weeks | 67.0± 1.8 | 65.2± 1.7 |  |  |  |

Data are presented as mean ± SEM. Linear mixed models were used to determine the effects of treatment (potato or rice), time (6- or 12-mo), period (1 or 2), the treatment*time interaction, and the treatment*period interaction with baseline included as a covariate on outcome measures. Body mass index (BMI); waist circumference (WC); hip circumference (HC); waist-to-hip ratio (WH); fat mass (FM); fat free mass (FFM). *Denotes statistical significance (*P*≤0.05).

**Supplementary Table 4.** Biomarkers of Glycemic Control, Cardiovascular Risk, and Lipid Profiles at Baseline, 6 and 12 Weeks After Potato and Rice Treatment Regimen.

| **Variable** | **Potato**  **(n=19)** | **Rice**  **(n=21)** | | **Treatment** | **Timepoint** | **Treatment-by- Timepoint** |
| --- | --- | --- | --- | --- | --- | --- |
| ***Glucose (mg/dL)*** |  |  | |  |  |  |
| Baseline | 141±6.2 | | |  |  |  |
| 6 Weeks | 144± 8.6 | | 144± 11.8 | 0.69 | 0.94 | 0.69 |
| 12 Weeks | 136± 9.8 | | 148± 13.7 |  |  |  |
| ***Insulin (mmol/L)*** |  | |  |  |  |  |
| Baseline | 21.0±0.9 | | |  |  |  |
| 6 Weeks | 20.4±2.1 | | 21.3±3.3 | 0.98 | 0.69 | 0.96 |
| 12 Weeks | 24.2±3.7 | | 24.3±1.7 |  |  |  |
| ***HOMA-IR*** |  | |  |  |  |  |
| Baseline | 6.33±0.46 | | |  |  |  |
| 6 Weeks | 7.11± 1.0 | | 7.71±3.32 | 0.74 | 0.69 | 0.94 |
| 12 Weeks | 8.23± 1.47 | | 8.80± 1.05 |  |  |  |
| ***HOMA-β*** |  | |  |  |  |  |
| Baseline | 87.0±6.2 | | |  |  |  |
| 6 Weeks | 85.9±10.1 | | 107±15.9 | 0.85 | 0.35 | 0.99 |
| 12 Weeks | 118±17.5 | | 147± 20.4 |  |  |  |
| ***OX-LDL (u/L)*** |  | |  |  |  |  |
| Baseline | 92.2±6.2 | | |  |  |  |
| 6 Weeks | 68.8±3.9 | | 70.6±5.7 | 0.73 | 0.69 | 0.94 |
| 12 Weeks | 60.7±4.5 | | 67.3±5.6 |  |  |  |
| ***CRP (μg/ml)*** |  | |  |  |  |  |
| Baseline | 0.08±0.02 | | |  |  |  |
| 6 Weeks | 0.15±0.04 | | 0.17±0.04 | 0.92 | 0.35 | 0.94 |
| 12 Weeks | 0.08±0.03 | | 0.08±0.03 |  |  |  |
| ***HbA1c (g/ml)*** |  | |  |  |  |  |
| Baseline | 2.61e^-5^±2.4e^-9^ | | | 0.85 | | |
| 12 Weeks | 2.73e^-5^±2.2e^-9^ | | 2.98 e^-5^±2.5 e^-9^ |  |  |  |
| ***Leptin (g/ml)*** |  | |  |  |  |  |
| Baseline | 2.44e^-8^±5.2e^-9^ | | |  |  |  |
| 6 Weeks | 2.10e^-8^±4.9e^-9^ | | 2.45e^-8^±5.2e^-9^ | 0.69 | 0.94 | 0.69 |
| 12 Weeks | 2.07e^-8^±4.7e^-9^ | | 2.79e^-8^±5.7e^-9^ |  |  |  |
| ***Adiponectin (g/ml)*** |  | |  |  |  |  |
| Baseline | 4.65e^-6^±5.5e^-7^ | | |  |  |  |
| 6 Weeks | 5.75e^-6^±1.1e^-6^ | | 5.48e^-6^±8.7e^-7^ | 0.94 | 0.69 | 0.96 |
| 12 Weeks | 6.56e^-6^±1.1 e^-6^ | | 6.31e^-6^±9.1e^-7^ |  |  |  |

| ***Total Cholesterol (mg/dL)*** |  |  |  |  |  |
| --- | --- | --- | --- | --- | --- |
| Baseline | 154±8.7 | |  |  |  |
| 6 Weeks | 167± 9.0 | 168± 16.3 | 0.98 | 0.79 | 0.96 |
| 12 Weeks | 156± 6.9 | 164± 12.2 |  |  |  |
| ***HDL (mg/dL)*** |  |  |  |  |  |
| Baseline | 51.0±3.4 | |  |  |  |
| 6 Weeks | 52.4± 3.5 | 50.1± 4.3 | 0.69 | 0.94 | 0.98 |
| 12 Weeks | 52.4± 3.5 | 53.1± 3.6 |  |  |  |
| ***Triglycerides (mg/dL)*** |  |  |  |  |  |
| Baseline | 132±13.1 | |  |  |  |
| 6 Weeks | 131± 15.1 | 142± 16.3 | 0.69 | 0.94 | 0.73 |
| 12 Weeks | 125± 14.1 | 151± 17.6 |  |  |  |
| ***Non-HDL*** |  |  |  |  |  |
| Baseline | 103±7.9 | |  |  |  |
| 6 Weeks | 114± 8.3 | 106± 10.1 | 0.96 | 0.94 | 0.79 |
| 12 Weeks | 104± 6.3 | 112± 11.4 |  |  |  |
| ***Total Cholesterol/ HDL Ratio*** |  |  |  |  |  |
| Baseline | 3.2±0.24 | |  |  |  |
| 6 Weeks | 3.4± 0.24 | 3.3±0.25 | 0.85 | 0.90 | 0.79 |
| 12 Weeks | 3.1± 0.26 | 3.3± 0.27 |  |  |  |
| ***LDL (mg/dL)*** |  |  |  |  |  |
| Baseline | 76.3±7.8 | |  |  |  |
| 6 Weeks | 88.3± 8.4 | 79.3± 9.2 | 0.96 | 0.92 | 0.91 |
| 12 Weeks | 78.7± 5.7 | 81.4± 9.8 |  |  |  |
| ***VLDL (mg/dL)*** |  |  |  |  |  |
| Baseline | 26.3±2.6 | |  |  |  |
| 6 Weeks | 26.0± 3.0 | 26.6±3.0 | 0.70 | 0.85 | 0.69 |
| 12 Weeks | 24.9± 2.8 | 30.0± 3.5 |  |  |  |

Data are presented as mean ± SEM. Linear mixed models were used to determine the effects of treatment (potato or rice), time (6- or 12-mo), period (1 or 2), the treatment*time interaction, and the treatment*period interaction with baseline included as a covariate on outcome measures. Homeostatic model of insulin resistance (HOMA-IR); homeostatic model of beta cell secretion (HOMA-*β*); endothelin-1 (ET-1); oxidized-low-density lipoprotein cholesterol (OX-LDL); C-reactive protein (CRP); glycated hemoglobin (HbA1c); high-density lipoprotein cholesterol (HDL); low-density lipoprotein cholesterol (LDL); very low-density lipoprotein cholesterol (VLDL). *Denotes statistical significance (*P*≤0.05). ^#^Denotes a tendency for statistical significance (*P*<0.1).

**Supplementary Table 5.** Hemodynamics and Arterial Stiffness at Baseline, 6 and 12 Weeks After Potato and Rice Treatment Regimen.

| **Variable** | **Potato**  **(n=19)** | **Rice**  **(n=21)** | **Treatment** | **Timepoint** | **Treatment-by-**  **Timepoint** |
| --- | --- | --- | --- | --- | --- |
| ***SBP (mmHg)*** |  |  |  |  |  |
| Baseline | 139 ± 4.9 | |  |  |  |
| 6 Weeks | 133 ± 3.8 | 134 ± 4.2 | 0.73 | 0.81 | 0.94 |
| 12 Weeks | 134 ± 4.2 | 137 ± 4.7 |  |  |  |
| ***DBP (mmHg)*** |  |  |  |  |  |
| Baseline | 80.1 ± 2.6 | |  |  |  |
| 6 Weeks | 77.9 ± 2.5 | 78.5 ± 2.3 | 0.69 | 0.99 | 0.77 |
| 12 Weeks | 76.5 ± 2.4 | 79.6 ± 2.6 |  |  |  |
| ***HR (bpm)*** |  |  |  |  |  |
| Baseline | 71.2±2.2 | |  |  |  |
| 6 Weeks | 70.6± 2.3 | 73.5±2.9 | 0.25 | 0.94 | 0.85 |
| 12 Weeks | 69.8± 2.9 | 73.7± 2.2 |  |  |  |
| ***MAP* (mmHg)** |  |  |  |  |  |
| Baseline | 99.7±3.0 | |  |  |  |
| 6 Weeks | 96.3±2.6 | 97.1±2.5 | 0.69 | 0.94 | 0.85 |
| 12 Weeks | 95.6±2.7 | 98.6± 2.9 |  |  |  |
| ***RPWV (MpS)*** |  |  |  |  |  |
| Baseline | 17.7±0.72 | |  |  |  |
| 6 Weeks | 17.8±0.87 | 18.0±0.79 | 0.94 | 0.94 | 0.94 |
| 12 Weeks | 17.9±0.85 | 18.0±0.80 |  |  |  |
| ***LPWV (MpS)*** |  |  |  |  |  |
| Baseline | 17.5±0.79 | |  |  |  |
| 6 Weeks | 17.4±0.92 | 17.9±0.85 | 0.94 | 0.94 | 0.69 |
| 12 Weeks | 17.9±0.89 | 17.6±0.81 |  |  |  |
| ***RABI*** |  |  |  |  |  |
| Baseline | 1.10±0.02 | |  |  |  |
| 6 Weeks | 1.11±0.02 | 1.13±0.02 | 0.98 | 0.69 | 0.85 |
| 12 Weeks | 1.14±0.02 | 1.14±0.02 |  |  |  |
| ***LABI*** |  |  |  |  |  |
| Baseline | 1.08±0.01 | |  |  |  |
| 6 Weeks | 1.10±0.02 | 1.12±0.02 | 0.77 | 0.70 | 0.89 |
| 12 Weeks | 1.12±0.02 | 1.16±0.02 |  |  |  |
| ***Aix (%)*** |  |  |  |  |  |
| Baseline | 27.4±2.0 | |  |  |  |
| 6 Weeks | 24.8±2.5 | 25.8±2.1 | 0.94 | 0.96 | 0.70 |
| 12 Weeks | 26.7±2.1 | 24.4±2.5 |  |  |  |

Data are presented as mean ± SEM. Linear mixed models were used to determine the effects of treatment (potato or rice), time (6- or 12-mo), period (1 or 2), the treatment*time interaction, and the treatment*period interaction with baseline included as a covariate on outcome measures. Systolic blood pressure (SBP); diastolic blood pressure (DBP); heart rate (HR); mean arterial pressure (MAP); pulse wave velocity-right side (RPWV); pulse wave velocity-left side (LPWV); ankle brachial index-right side (RABI); ankle brachial index-left side (LABI); augmentation index (Aix); meters per second (MpS). *Denotes statistical significance (*P*≤0.05). ^#^Denotes a tendency for statistical significance (*P*<0.1).

**Supplementary Table 6.** Vascular Assessments of Endothelial-Mediated Vasodilation at Baseline, 6 and 12 Weeks After Potato and Rice Treatment Regimen

| **Variable** | | | **Potato**  **(n=19)** | **Rice**  **(n=21)** | **Treatment** | **Timepoint** | **Treatment-by-**  **Timepoint** |
| --- | --- | --- | --- | --- | --- | --- | --- |
| ***Resting Diameter (mm)*** |  | | |  |  |  |  |
| Baseline | | 4.28±0.147 | | |  |  |  |
| 6 Weeks | | 4.31±0.150 | | 4.24±0.133 | 0.69 | 0.94 | 0.85 |
| 12 Weeks | | 4.28±0.141 | | 4.25± 0.139 |  |  |  |
| ***Resting BF (mL/min)*** | |  | |  |  |  |  |
| Baseline | | 123±18.7 | | |  |  |  |
| 6 Weeks | | 107±16.0 | | 102±15.1 | 0.94 | 0.90 | 0.99 |
| 12 Weeks | | 113±17.5 | | 108±10.8 |  |  |  |
| ***Max Dilation (mm)*** | |  | |  |  |  |  |
| Baseline | | 4.58±0.150 | | |  |  |  |
| 6 Weeks | | 4.62±0.149 | | 4.58±0.142 | 0.62 | 0.71 | 0.85 |
| 12 Weeks | | 4.60±0.145 | | 4.51± 0.155 |  |  |  |
| ***Time to Peak (sec)*** | |  | |  |  |  |  |
| Baseline | | 43.6±2.41 | | |  |  |  |
| 6 Weeks | | 45.2±2.25 | | 42.3±2.35 | 0.69 | 0.90 | 0.99 |
| 12 Weeks | | 46.5±2.95 | | 43.7±2.40 |  |  |  |
| ***Change in Diameter (mm)*** | |  | |  |  |  |  |
| Baseline | | 0.303±0.0233 | | |  |  |  |
| 6 Weeks | | 0.306±0.0220 | | 0.306±0.0219 | 0.73 | 0.69 | 0.77 |
| 12 Weeks | | 0.297±0.0230 | | 0.262±0.0259 |  |  |  |
| ***FMD (%)*** | |  | |  |  |  |  |
| Baseline | | 7.28±0.665 | | |  |  |  |
| 6 Weeks | | 7.30±0.609 | | 7.31±0.600 | 0.73 | 0.69 | 0.77 |
| 12 Weeks | | 7.17±0.646 | | 6.32±0.694 |  |  |  |
| ***Shear Rate (AUC)*** | |  | |  |  |  |  |
| Baseline | | 33891±2724 | | |  |  |  |
| 6 Weeks | | 33554±2915 | | 34065±3510 | 0.90 | 0.94 | 0.94 |
| 12 Weeks | | 33449±2366 | | 35473±2901 |  |  |  |
| ***FMD/Shear*** | |  | |  |  |  |  |
| Baseline | | 0.000229±0.0000213 | | |  |  |  |
| 6 Weeks | | 0.000258±0.0000353 | | 0.000245±0.0000271 | 0.69 | 0.47 | 0.90 |
| 12 Weeks | | 0.000227±0.0000220 | | 0.000190±0.0000202 |  |  |  |

Data are presented as mean ± SEM. Linear mixed models were used to determine the effects of treatment (potato or rice), time (6- or 12-mo), period (1 or 2), the treatment*time interaction, and the treatment*period interaction with baseline included as a covariate on outcome measures. Resting blood flow (Resting BF); flow-mediated dilation (FMD); flow-mediated dilation/ shear rate (FMD/Shear). *Denotes statistical significance (*P*≤0.05). ^#^Denotes a tendency for statistical significance (*P*<0.1).


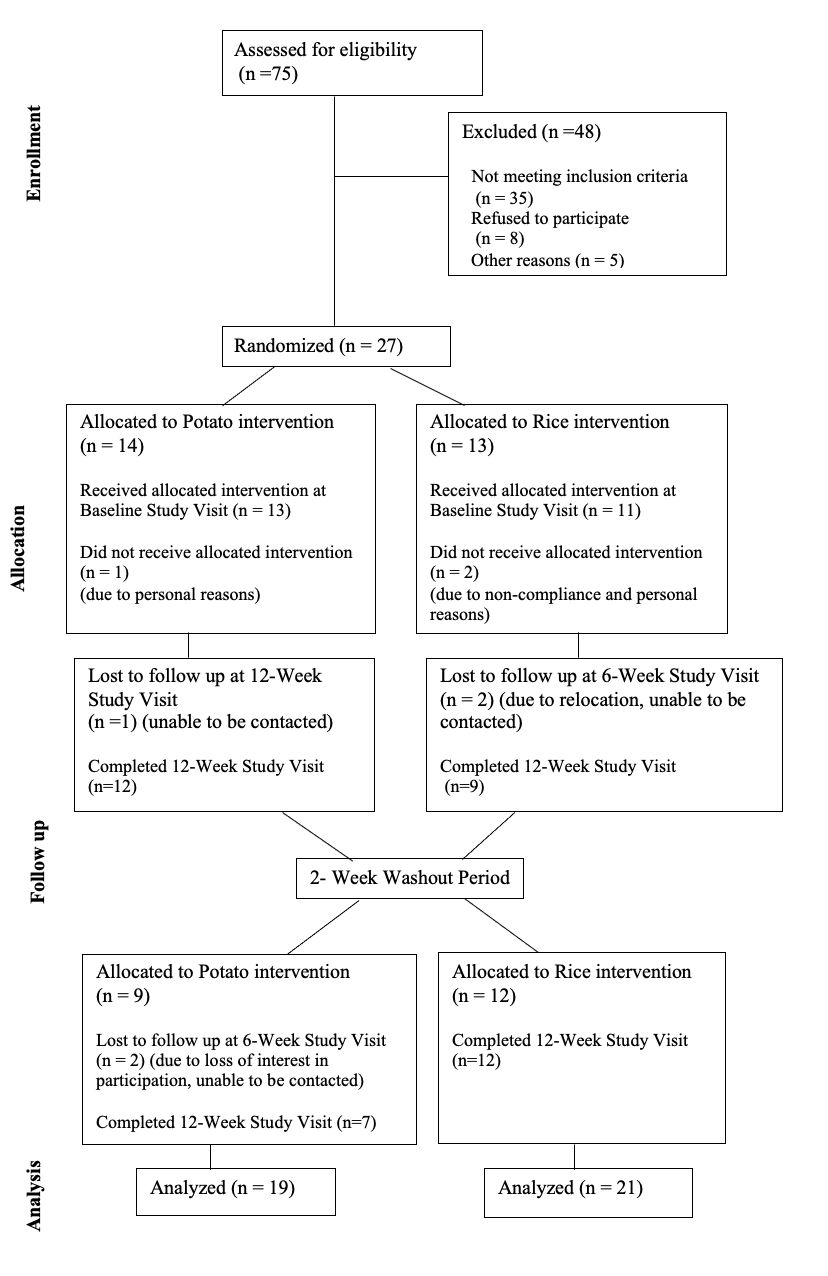
**Supplementary Figure 1.** CONSORT Flowchart of Study Enrollment


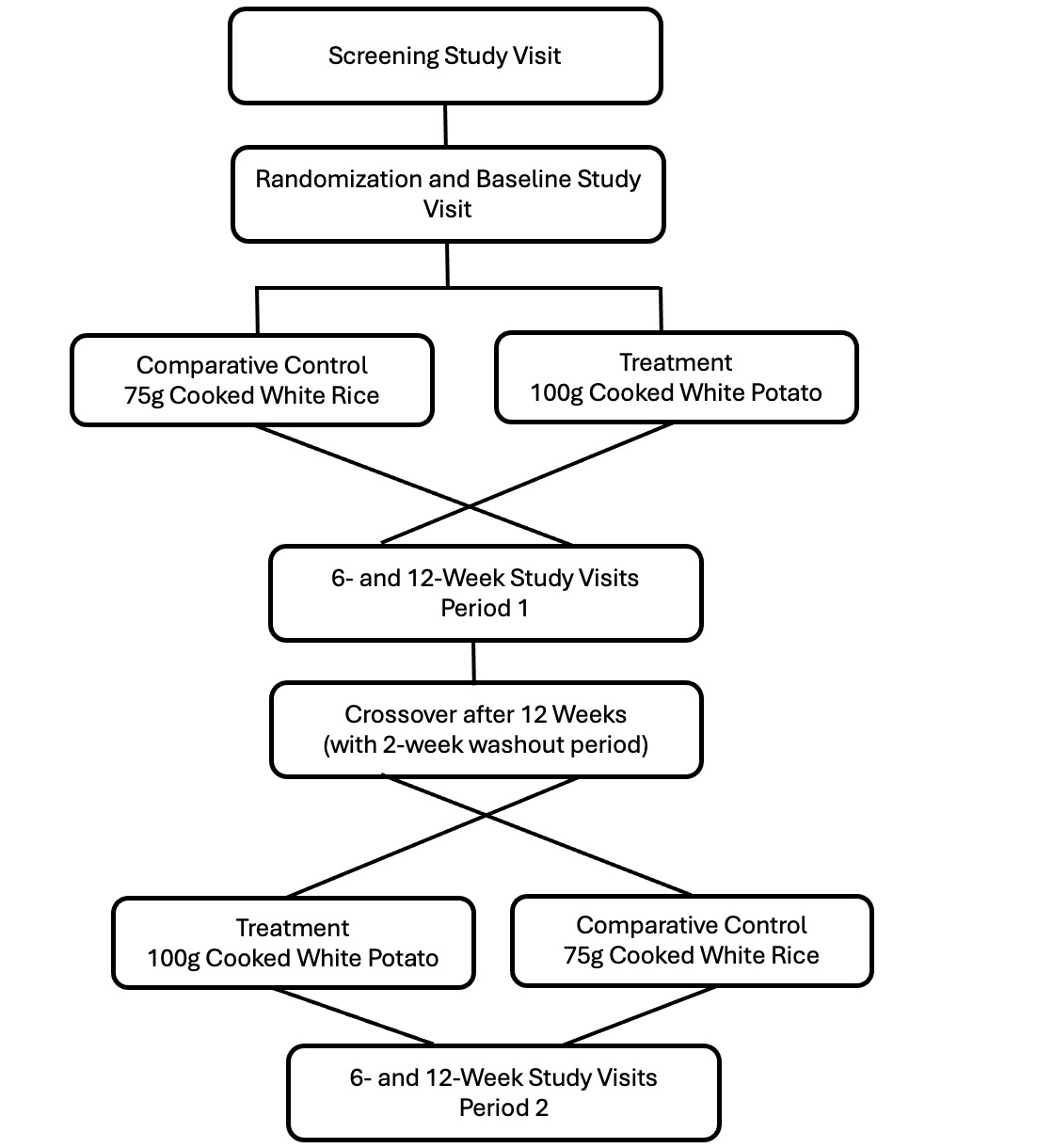


**Supplementary Figure 2.** Study Design Flowchart
